# An Overview of First 100 Cardiac Surgery Cases at a Newly Developed Satellite Center in Sukkur, Pakistan

**DOI:** 10.7759/cureus.8490

**Published:** 2020-06-07

**Authors:** Kashif Zia, Ali R Mangi, Syed Minhaj, Khuzaima Tariq, Fazal Rabbi, Muhammad Musharaf, Muhammad Asad Bilal Awan, Rizwan A Memon, Asif R Rathore, Pervaiz A Chaudry

**Affiliations:** 1 Cardiac Surgery, National Institute of Cardiovascular Diseases, Karachi, PAK; 2 Cardiac Surgery, College of Physicians and Surgeons Pakistan, Karachi, PAK

**Keywords:** cardiovascular diseases, cardiac surgery, pakistan, satellite center, nicvd, sukkur, mics, opcab, off-pump coronary artery bypass (opcab), on-pump coronary artery bypass (oncab)

## Abstract

Introduction

The aim of this research is to evaluate the in-hospital and early outcomes of the first 100 adult cardiac surgeries performed at a newly developed satellite center in Sukkur, Pakistan.

Methods

This is an audit of the first 100 adult cardiac surgeries performed at a newly developed satellite center of the National Institute of Cardiovascular Diseases (NICVD) at Sukkur, Pakistan, from March 2018 to November 2018 with 12 months of post-operative follow-up. Patients were offered off-pump coronary artery bypass (OPCAB), on-pump coronary artery bypass (ONCAB), mitral valve replacement (MVR), aortic valve replacement (AVR), minimally invasive cardiac surgery (MICS), and congenital adult congenital heart disease (ACHD) procedures by expert faculty of NICVD with a minimum of five years of post-fellowship experience.

Results

The mean age was 47.11 ± 14.6 years, with a male predominance of 77%. Hypertension and smoking were the most common risk factors that were observed in 32% and 33%, respectively, followed by diabetes and dyslipidemia with a frequency of 20% and 9%, respectively. The mean EuroSCORE (European System for Cardiac Operative Risk Evaluation) II for this patient cohort was 1.165 ± 0.50, with a maximum score of 2.3 in one patient. Out of 100 procedures, 51 were ONCAB, 19 were OPCAB, 16 were MVR, three were AVR, nine were ACHD, and two were MICS. Survival status post-operative as well as after one year was 100%. The frequency of post-operative bleeding was 7%, mean post-operative mechanical ventilation time was 213 ± 273 hours, and in-hospital stay was 5.41 ± 0.165 days. Lost to follow-up at one year was 4% (four). During the follow-up assessment, 39.5% of the patients had complained of mild-to-moderate intensity retrosternal pain and 4.2% had superficial surgical site infection of the sternal wound. A significant improvement in functional class was observed in 38.5% of patients, whereas 4.2% (four) had a significant drop in functional class post-operatively.

Conclusion

Providing tertiary care and early cardiac surgical facility to the people of Sukkur at their doorstep, in a newly developed satellite center, has resulted in improved outcomes, early quality treatment facility, and avoidance of long travel time.

## Introduction

The incidence of cardiovascular diseases has markedly increased in Pakistan for the last few years, and the hidden portion of the iceberg is growing day by day. Even the latest reported incidence of coronary artery disease and rheumatic heart disease requiring surgery in Pakistan dates back a decade [1-4].

Cardiac surgery is a field in which there is a high concentration of patients in the metropolitan area because the surgery is mainly carried out in medical institutions located in the metropolitan area. Due to this, policies must be developed in order to enhance the quality of healthcare and the geographical accessibility for patients residing in remote regions [5].

The need to introduce such centers at areas of the Sindh province came up due to high flow of cardiac patients traveling to Karachi for cardiac intervention or surgery, with more than 400 patients waiting for cardiac surgery at the National Institute of Cardiovascular Diseases (NICVD), as per the Karachi Clinics Registry, before the start of Sukkur, Larkana, and Tando Mohammad Khan (TMK) surgical centers. This figure has been markedly brought down now, so have earlier admissions, reduced traveling time, regular follow-up visits, and cost burden to the patient. The idea of establishing regional cardiac surgery centers has been reported in South Korea to meet the patient’s requirements [5]. Also, the training requirement of more and more young cardiac surgeons can be effectively completed as the United States reports shortage of cardiothoracic surgeons by 2020 [6].

The inauguration of this 300-bed state-of-the-art satellite center in Sukkur took place on February 24, 2018. This center provides services such as modern and well-equipped 24/7 cardiac emergency, Cath Lab, coronary care unit, consulting clinics, advanced diagnosis, adult and pediatric cardiology, echocardiography, coronary artery angioplasty, angiographies, and cardiac surgical facility.

With the inauguration of cardiac surgery equipped satellite facilities by NICVD at Sukkur, Larkana, and TMK, there has been a lot of ease for people to get prompt cardiac care.

The first cardiac surgery at NICVD Sukkur took place in March 2018. Since then, this center has been providing complete cardiac surgical coverage to the population of Sukkur city and surrounding areas. To date, more than 500 cases have been operated at Sukkur, whereas this study only reports the starting of this newly developed center.

## Materials and methods

This retrospective observational study is an audit of the first 100 cases of cardiovascular surgeries performed at a newly developed satellite center of the NICVD at Sukkur from March 2018 to November 2018 with 12 months of post-operative follow-up.

Patients selected for any procedure met the American Heart Association guidelines criteria and had undergone heart team approval as per hospital policy before undergoing cardiac surgery. Pre-operative informed consent was taken from the patient and family. The European System for Cardiac Operative Risk Evaluation (EuroSCORE) II online calculator was used for risk stratification of patients [7]. All the patients electively admitted for surgery were offered guideline-directed general pre-operative measures to limit peri-operative complications [8].

Patients were offered off-pump coronary artery bypass (OPCAB), on-pump coronary artery bypass (ONCAB), mitral valve replacement (MVR), aortic valve replacement (AVR), minimally invasive cardiac surgery (MICS), and congenital procedures by expert faculty of NICVD with a minimum of five years of post-fellowship experience.

OPCAB was performed using OctoBase® sternal retractor, Octopus® stabilizer, Urchin positioner®, and AccuMist® Blower (all by Medtronic, Minneapolis, MN, USA). OPCAB myocardial protection was performed using intracoronary shunts size ranging from 1.0 to 2.0 mm with the left internal mammary artery (LIMA) to the left anterior descending artery (LAD) as the first strategy. ONCAB and valvular procedures were performed through median sternotomy with target activated clotting time above 480 seconds and myocardial protection using St. Thomas cardioplegia delivered antegrade (graft plegia was preferred by few surgeons) or retrograde with topical cooling. Minimally invasive atrial septal defect (ASD) closure was performed using the same technique as described in our previous publication on double valve replacement with central cannulation [9].

After discharge, all the patients were scheduled for routine follow-up visits at the cardiac surgical clinic of NICVD Sukkur at least four times, i.e., after one week, one month, six months, and one year after the procedure. They were also contacted by cell phone as a reminder for their follow-up visit. Early post-operative echocardiography was performed on the first post-operative day and before discharge, whereas follow-up echocardiography was performed in all patients after six months of the procedure. Patients requiring vitamin K antagonists (VKA) were managed in the INR (international normalized ratio) clinic. All patients were referred to the rehabilitation center for early recovery. Patients requiring emergent medical attention were asked to report to the NICVD emergency room or clinic at any time.

During their hospital stay, the patients were kept under observation, and in-hospital (30 days post-operative) outcomes were assessed, which included mortality, morbidity, length of stay, ventilation time, post-operative bleeding, reoperation, and re-intubation.

At one-year follow-up, survival status, complaints, infection (superficial or deep), function class, echocardiographic assessment, and recurrence of disease were assessed. Data analysis was carried out using SPSS Statistics for Windows, Version 21.0 (IBM Corp., Armonk, NY, USA).

## Results

The mean age of the first 100 patients’ undergone cardiovascular surgeries at this satellite center was 47.11 ± 14.6 years, with a male predominance of 77%. Hypertension and smoking were the most common risk factors which were observed in 32% and 33%, respectively, followed by diabetes and dyslipidemia with a frequency of 20% and 9%, respectively. The mean EuroSCORE II for this patient cohort was 1.165 ± 0.50, with a maximum score of 2.3 in one patient. Mean ejection fraction (EF) was 56.33 ± 6.96%. Distribution of baseline demographic and clinical characteristics of the patients by gender are presented in Table 1.

**Table 1:** Baseline demographic and clinical characteristics of the patients by gender NYHA, New York Heart Association; EuroSCORE, European System for Cardiac Operative Risk Evaluation

Characteristics	Males	Females
Total	77	23
Age, years	50 ± 13	36 ± 13
Weight, kg	73 ± 14	59 ± 19
NYHA classification		
I	3.9% (3)	6.5% (5)
II	42.9% (33)	6.5% (5)
III	44.2% (34)	15.6% (12)
IV	9.1% (7)	1.3% (1)
Co-morbidities		
Diabetics	24.7% (19)	1.3% (1)
Hypertension	40.3% (31)	1.3% (1)
Dyslipidemia	10.4% (8)	1.3% (1)
Smoking	41.6% (32)	1.3% (1)
Cardiogenic shock	6.5% (5)	2.6% (2)
Ejection fraction	56.32 ± 7.14	56.35 ± 6.50
EuroSCORE II	1.23 ± 0.49	0.97 ± 0.52

**Figure 1:**
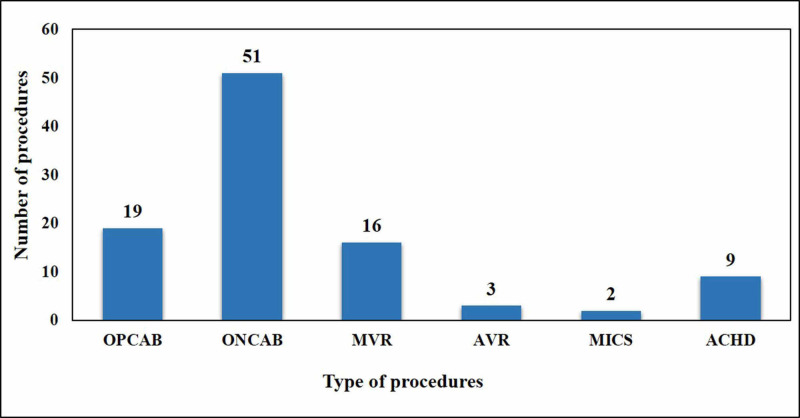
Frequency distribution of types of first 100 procedures performed at Sukkur satellite center OPCAB, off-pump coronary artery bypass; ONCAB, on-pump coronary artery bypass; MVR, mitral valve replacement; AVR, aortic valve replacement; MICS, minimally invasive cardiac surgery; ACHD, adult congenital heart disease procedures

A majority of the smokers, 36.3% (12/33), had a history of more than one pack a year. These patients underwent breathing exercises, chest physiotherapy, and spirometry before surgery and pulmonary function test assessment for the prediction of future outcomes. Among diabetic patients, 65% (13/20) had uncontrolled insulin-dependent diabetes mellitus, whereas 15% (3/20) had an incidental diagnosis of diabetes after pre-operative routine laboratory investigations. Mean HbA1c of diabetic patients was 9.85 ± 2.0%; therefore, surgery was considered once this figure was brought around 7.5. Perioperative dose calculated insulin infusion was used in these patients during the course of surgery with guideline-directed target blood sugar. Frequency distribution of the types of procedures is presented in Figure 1.

A total of 70 coronary artery bypass grafting (CABG) surgeries were performed out of which 7 were operated as emergency cases due to signs of cardiogenic shock, whereas the rest of the patients were electively operated and admitted through the outpatient department. Nineteen (27.1%) of CABG surgeries were OPCAB, whereas the remaining 72.9% (51/70) were conventional ONCAB surgeries. Our primary goal remained completely revascularization, with the LIMA as a preferred conduit for the LAD to improve survival benefit for the patient. We achieved a mean number of distal grafts as 3.01 ± 0.577, including 11 patients in the CABG group who had two distal targets for anastomosis. Detailed frequency of types of procedures against the subsequent diagnosis and post-operative outcomes are presented in Table 2.

**Table 2:** : Diagnosis, procedures, and post-operative outcomes of the first 100 procedures performed at Sukkur satellite center CABG, coronary artery bypass grafting; CVA, cerebrovascular accident

Diagnosis and procedures	Frequency
CABG	
Triple-vessel coronary artery disease	52
Two-vessel coronary artery disease	8
Left main with triple-vessel coronary artery disease	6
Left main with two-vessel coronary artery disease	4
Aortic valve replacement (mechanical valves)	
Severe symptomatic aortic regurgitation	2
Severe symptomatic aortic stenosis	1
Mitral valve replacement (mechanical valves)	
Severe symptomatic mitral stenosis	9
Severe symptomatic mixed mitral disease	7
Minimally invasive and conventional atrial septal defect closure	
Secundum atrial septal defect	8
Conventional ventricular septal defect closure	
Ventricular septal defect	2
Pulmonary valvotomy	
Isolated pulmonary stenosis	1
Post-operative outcomes	
Mortality	0
CVA	1
Low cardiac output state	1
Renal dysfunction	1
Post-operative bleeding	7
Reopening	3
Re-intubation	2
Ventilation time (hours)	213 ± 273
Length of stay (days)	5.41 ± 0.165

Out of 70 patients, 65 (93%) had LIMA anastomosed to LAD, in 4 patients LIMA was not used considering co-morbidities and life expectancy, and 1 patient had an operative injury to LIMA. MVR surgeries were performed in 16 patients, AVR in 3, adult congenital heart surgery in 11, out of which 2 patients were offered minimally invasive right anterior mini-thoracotomy ASD closure.

The frequency of surgically significant post-operative bleeding was observed in seven (7%) of the patients, out of which three patients were surgically managed, whereas the rest of the patients had disturbed coagulation profile and were therefore managed on medical therapy. Mean post-operative mechanical ventilation time remained 213 ± 273 hours, which also included total mechanical ventilation time for complicated patients requiring prolonged ventilation and those who had re-intubation. In-hospital stay remained 5.41 ± 0.165 days, with 0% in-hospital mortality.

Three patients underwent immediate reoperation. One was diagnosed with perioperative cardiac tamponade, and, therefore, emergent resternotomy in the intensive care unit (ICU) was performed to relieve the tamponade; afterward the patient was moved to the operating room. Two patients had perioperative massive bleeding, which were managed in the operating room

Intra-aortic balloon pump (IABP) was electively placed in six patients undergoing CABG, whereas one patient had an emergent IABP placement because of post-operative low cardiac output state (LCOS). The patient who developed LCOS underwent re-intubation as well because of secondary respiratory failure as well. Along with that, another patient was re-intubated who had post-operative renal dysfunction requiring dialysis. One patient had post-operative ischemic stroke. All of these complicated patients requiring a multidisciplinary approach were managed by intensivists, nephrologists, and neurologists.

Lost to follow-up at one year was 4% (four), and follow-up of the remaining 96 (96%) patients was completed successfully. During the follow-up assessment, 39.5% (38/96) patients had complained of mild-to-moderate intensity retrosternal pain with numbness over the left parasternal area, 4.2% (four) had superficial surgical site infection of the sternal wound, and one patient had a sternal dehiscence and underwent rewiring for sternal closure. A significant improvement in functional class was observed in 38.5% (37/96) of the patients, whereas 4.2% (four) had a significant drop in functional class, for which all of these patients had complications during the perioperative period. One of the patients had dropped in EF from 45% to 35% due to early graft occlusion and underwent percutaneous coronary intervention for disease recurrence. Survival status at one-year follow-up remained 100%. On-year follow-up status of patients is presented in Table 3.

**Table 3:** One-year follow-up status of the first 100 procedures performed at Sukkur satellite center SSI, surgical site infection

One-year follow-up	
N	96
Survival status	100% (96)
Complaints	
Pain	39.6% (38)
Cough	36.5% (35)
Serous discharge	24% (23)
Muscle cramps	18.8% (18)
Sleeplessness	16.7% (16)
Anxiety	12.5% (12)
Immobility	2.1% (2)
Infection	
Superficial (SSI) sternal	4.2% (4)
Superficial (SSI) leg	3.1% (3)
Deep sternal wound infection	0% (0)
Sternal dehiscence	1% (1)
Function class	
Deterioration	4.2% (4)
Improvement	38.5% (37)
Echocardiographic assessment	
Deterioration ejection fraction	34.4% (33)
Severe pericardial effusion	1% (1)
Structural valvular deterioration	4.2% (4)
Paravalvular leak	0% (0)
Recurrence of disease	1% (1)

## Discussion

Closed heart operations in Pakistan began by the native surgeons in the late 1950s at a few centers in Karachi, Lahore, and Rawalpindi. But open-heart operations began by Dr. Donald Edward Bowes, a Canadian born missionary surgeon in 1967-1968 at United Christian Hospital Lahore [10]. Regular civilian cardiac surgical program began in Pakistan with the establishment of the NICVD at Karachi in the early 1970s [11]. Since then, NICVD has remained the center of excellence to provide quality care to cardiac patients.

In the Third World, cardiac patients often suffer from not only the nature of their illnesses but also the insufficiency of the facilities available [12]. There has been a marked rise in the patients requiring cardiac surgery, and Pakistan has a very limited number of government-funded centers to timely operate on these patients. Therefore, the only way out remained to enlist these patients and make them wait for their surgery. This resulted in a lot of morbidity, out-of-hospital cardiac arrest (OHCA) events, and mortality in these patients waiting for their turn to undergo coronary bypass or valve-related surgery because of lack of rural cardiac facilities. Therefore, expanding the cardiac surgical coverage remained a primary focus for health care providers. Till date, more than 500 successful cardiac surgeries have been performed at NICVD Sukkur. With an aim to minimize the time required by a patient to avail early cardiac care, the team of NICVD is moving forward to provide a near-to-door cardiac facility across Pakistan.

We have faced a huge burden of diabetics, hypertension, and smoking in the Sukkur population, predicting a higher risk for adverse outcomes. But we have remained successful to prove that timely surgical intervention, adequate pre-operative management, and regular follow-up are keys to successful outcomes at a newly developed cardiac surgery institute. Institutional results are essential to enable surgeons to determine whether international data are in keeping with local findings [13]. NICVD Sukkur being first of its kind government-funded state of the art cardiac surgical facility has demonstrated impressive surgical results compared with reliable Western standards.

NICVD being a non-private fully funded government organization regards maximum post-operative management protocols with zero financial burdens on families. This facility has provided all advanced resources such as standardized medications, IABP, continuous renal replacement therapy machine, automatic implantable cardiac defibrillators, ventricular assist devices, and extracorporeal membrane oxygenation. This means that patients who suffer from a poor post-operative recovery did receive the most optimal possible support. This should encourage other hospitals in the developing world to aspire to high levels of care.

## Conclusions

With an increasing incidence of patients requiring cardiac surgeries, the inauguration of new cardiac surgical centers is a necessity in Pakistan. Establishment of the Sukkur satellite center has allowed for the provision of quality cardiac surgical care. Despite being a newer unit, it experienced faculty backup has resulted in enormous pre- and post-operative management with minimum reported complications and zero mortality in the first 100 cases during the in-hospital period.
